# Minimal Invasive Decompression for Lumbar Spinal Stenosis

**DOI:** 10.1155/2012/645321

**Published:** 2012-04-04

**Authors:** Victor Popov, David G. Anderson

**Affiliations:** ^1^Department of Orthopaedic Surgery, Thomas Jefferson University and Rothman Institute, Philadelphia, PA 19107, USA; ^2^Department of Neurologic Surgery, Thomas Jefferson University and Rothman Institute, Philadelphia, PA 19107, USA

## Abstract

Lumbar spinal stenosis is a common condition in elderly patients and may lead to progressive back and leg pain, muscular weakness, sensory disturbance, and/or problems with ambulation. Multiple studies suggest that surgical decompression is an effective therapy for patients with symptomatic lumbar stenosis. Although traditional lumbar decompression is a time-honored procedure, minimally invasive procedures are now available which can achieve the goals of decompression with less bleeding, smaller incisions, and quicker patient recovery. This paper will review the technique of performing ipsilateral and bilateral decompressions using a tubular retractor system and microscope.

## 1. Introduction

 Lumbar spinal stenosis remains the most common indication for spinal surgery in elderly patients [1–8]. Lumbar spinal stenosis is a pathologic state where the dural sac and nerve roots are compressed by a combination of degenerative features including bulging of the intervertebral discs, hypertrophy of the facet joints, and thickening/buckling of the ligamentum flavum. The clinical symptoms of this condition include back and leg pain, muscular weakness, sensory disturbance, and/or problems with ambulation [9]. Although the severity of clinical symptoms varies widely, some patients may experience disabling symptoms which required medical intervention [1–5, 10, 11]. The traditional surgical approach for lumbar stenosis has been to perform a wide, bilateral decompressive laminectomy along with resection of the medial portion of the facet joints to decompress the affected neural elements [7, 8, 12, 13]. Although this approach can successfully alleviate nerve compression symptoms, there are drawbacks of the open approach, including amount of soft tissue dissection, blood loss, postoperative pain, and the potential for iatrogenic instability of the spinal segment [14]. These concerns are magnified when treating an elderly fragile patient.

The use of a tubular retractor system for lumbar surgery was popularized by Foley and Smith [15]. As experience has grown with this surgical approach, surgeons are routinely treating patients with lumbar stenosis using a combination of a tubular retractor system and an operative microscope. This approach requires less soft tissue destruction compared to an open lumbar decompression [9, 16, 17]. As a result, the surgeon can expect less bleeding, less postoperative pain, and a reduced risk of iatrogenic instability. Surgery with a tubular retractor system is especially beneficial in elderly patients where there are concerns regarding the physiologic stress and risks of a traditional open surgical approach [2].

 This paper will review the operative techniques for treating lumbar stenosis with a tubular retractor system and operative microscope.

## 2. Surgical Setup

**Figure 1:**
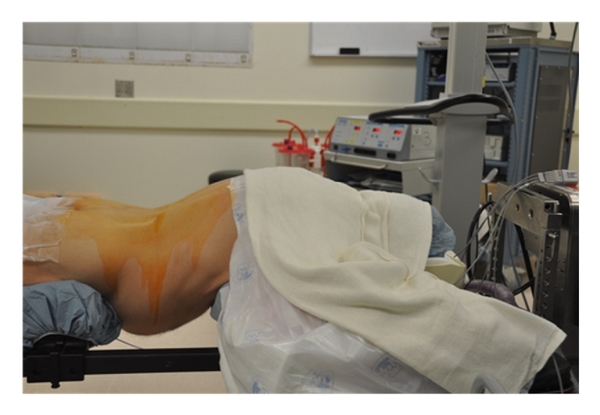
Positioning of the patient in the prone position on a radiolucent operative table.

 The procedure is typically performed under general anesthesia, although epidural or spinal anesthesia can be used according to surgeon preference. Prophylactic antibiotics and lower extremity compression stockings are provided at the initiation of the procedure. The patient is positioned prone on a radiolucent spinal frame which allows decompression of the abdomen and access for fluoroscopic imaging (Figure 1).

## 3. Surgical Approach

**Figure 2:**
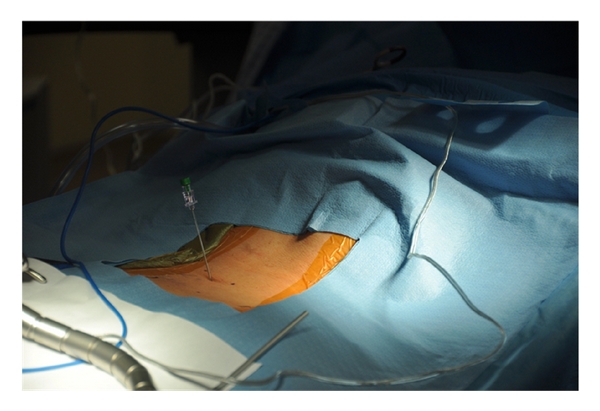
demonstrates a spinal needle introduced at the proposed location of the surgical incision.

**Figure 3:**
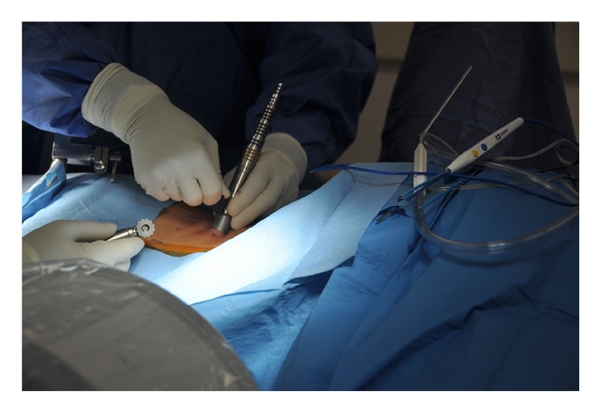
Serial dilation of the soft tissue corridor and placement of the correct length tubular retractor.

**Figure 4:**
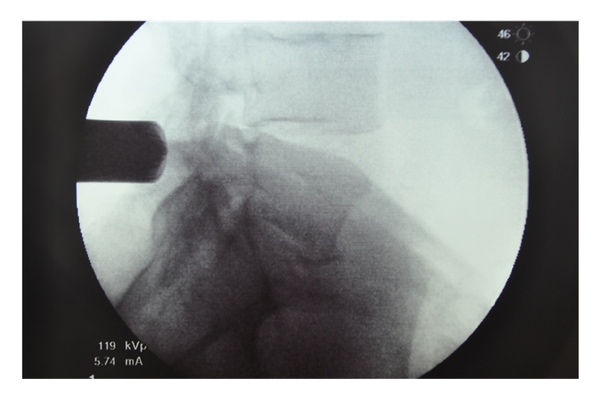
shows the position of the tubular retractor using lateral fluoroscopy.

 After a sterile prep and drape, the location of the spinous processes and iliac crests are marked out on the skin as a guide when localizing the surgical incision. A spinal needle is introduced at the proposed location of the surgical incision, and lateral C-arm fluoroscopy is used to check the position of the needle relative to the site of the neural compression (Figure 2). After confirming correct localization of the needle, the surgical incision is made lateral to the spinous processes. For ipsilateral decompression, the skin incision should be placed about 2 cm lateral to the midline, while bilateral decompression requires an incision about 3 cm lateral to the midline to allow angulation of the tubular retractor to reach the contralateral side of the spinal canal. The length of the incision should be equal to the diameter of the tubular retractor to be used. The authors prefer to use an 18–20 mm outer diameter tubular retractor when performing a decompressive procedure for lumbar stenosis. The thoracolumbar fascia should be sharply incised in line with the skin incision. Next, a small Cobb elevator is placed through the incision down to the spinal lamina, and subperiosteal elevation of muscle tissues away from the lamina is performed. Serial dilation of the soft tissue corridor is carried out followed by placement of the correct length tubular retractor (Figure 3). It is important to be sure that the tubular retractor is firmly seated against the bone of the lamina before securing the tube with a table-mounted retractor holder. Next, a lateral fluoroscopic image is used to confirm correct localization of the tubular retractor (Figure 4).

**Figure 5:**
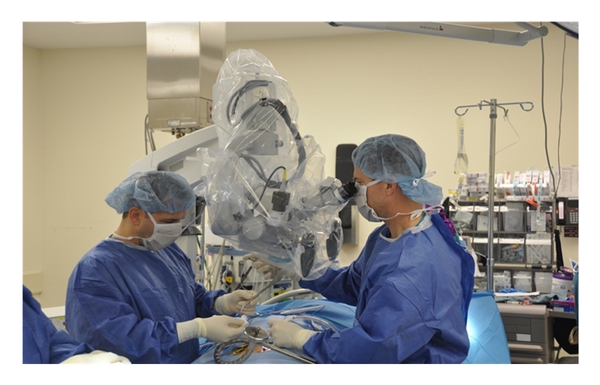
shows operative microscope used to visualize the operative field.

**Figure 6:**
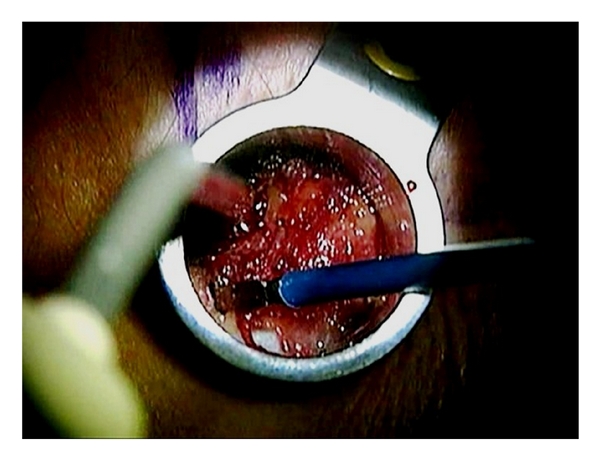
Residual soft tissues are removed with electrocautery to expose the lamina and medial edge of the facet joint.

 The operative microscope is then used to visualize the operative field at the base of the tubular retractor (Figure 5). Any residual soft tissues are removed with electrocautery to expose the lamina and medial edge of the facet joint prior to proceeding (Figure 6).

## 4. Ipsilateral Decompression

**Figure 7:**
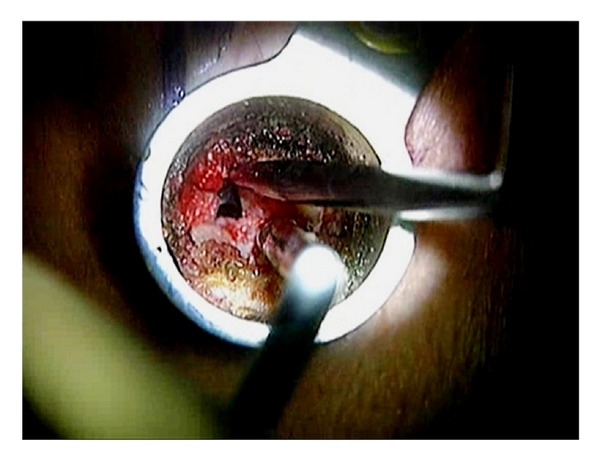
A curved curette is used to separate the ligamentum flavum from the undersurface of the lamina.

**Figure 8:**
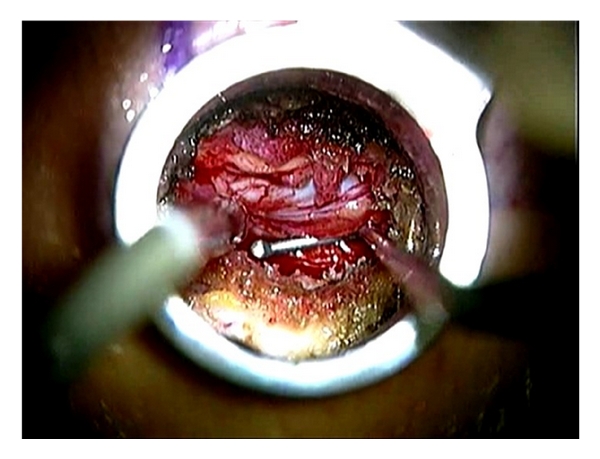
A ball-tipped probe is used for the palpation during and at the end of the decompression procedure.

 A curved curette is used to separate the ligamentum flavum from the undersurface of the lamina (Figure 7). Then, the ipsilateral lamina is removed with a Kerrison rongeur or high-speed drill/burr. The laminotomy should progress to the cranial edge of the ligamentum flavum. If only the ipsilateral side requires decompress, the ligamentum flavum is then removed. However, if bilateral decompression is required (see below), the ligamentum flavum is left intact until after the drilling maneuver has been completed across to the contralateral side. After removal of the ligamentum flavum, the pedicle (as a landmark) is examined by palpation with a ball-tipped probe for identification of the spinal pathology, the medial portion of the facet joint is trimmed as needed to achieve decompression of the lateral recess. The overlying inferior articular process may need to be thinned with a high-speed drill/burr, but care should be taken to preserve adequate bone stock in this region so as to reduce the risk of an iatrogenic fracture. A curved tip kerrison rongeur is used to undercut the lateral recess while preserving the overlying bone stock of the facet complex. The ipsilateral foramen is decompressed by resecting the superior tip of the superior articular process as needed to decompress the exiting nerve root. The disc space is examined, and any herniated disc fragments are removed. Finally, the adequacy of decompression is confirmed with the use of a ball-tipped probe (Figure 8). Hemostasis of the wound is then achieved prior to removal of the tubular retractor system.

## 5. Contralateral Decompression

When a bilateral decompression is required, the tube is angled (wanded) to the contralateral side after the ipsilateral lamina has been opened (but prior to resection of the ligamentum flavum). The operative table can be angled away from the surgeon and the operative microscope repositioned to provide visualization at the base of the spinous process. Next, a high-speed drill/burr is used to drill away the ipsilateral base of the spinous process dorsal to the ligamentum flavum. Bone bleeding in this region is controlled with bone wax. A small currette is used to separate the ligamentum flavum from the contralateral lamina, and the drilling is continued through the contralateral lamina until the contralateral facet joint is reached. It is important to note that a bone bridge is left connecting the contralateral base of the spinous process and dorsal surface of the contralateral lamina. The “internal laminectomy” is continued along the contralateral lamina until the contralateral facet joint is reached. The medial portion of the contralateral facet is thinned until it can be successfully undercut with a Kerrison Rongeur to adequately decompress the lateral recess and foraminal area. After the drilling maneuver is completed, the ligament flavum is separated from its bony attachments and removed. Under direct visualization of the neural elements, any remaining bony or ligamentous compression is alleviated. The adequacy of the decompression is confirmed with a ball-tipped probe. After completion of the contralateral decompression, the tubular retractor is adjusted (wanded) back to the ipsilateral side, and the decompression of the ipsilateral side is completed as described above.

## 6. Wound Closure and Aftercare

 The fascia, subcutaneous tissues, and skin are closed in a routine fashion. A skin sealant is placed along the skin edges to allow early showering. The subcutaneous tissues are injected with a long-acting local anesthetic to reduce incisional pain, followed by placement of a small dressing.

 Patients are mobilized after recovery from anesthesia and discharged on the same day as surgery (in most cases). Early return to ambulation and normal activities of daily living is encouraged. Pain management is generally provided by either a mild oral narcotic or an over-the-counter analgesic depending on the preferences of the patient. Rehabilitation with core muscle stabilization and aerobic activities are encouraged in the early postoperative period.

## 7. Complications

 Although the list of potential complications with tubular decompression is no different from traditional open surgery, the rate of certain complications is significantly reduced. For instance, blood loss, wound infection, iatrogenic instability, and medical deterioration following lumbar decompression using a tubular retractor system are lower compared to open laminectomy [9, 16, 17].

 Dural laceration (incidental durotomy) may be managed with either suture repair or dural sealants depending on the location, size, and severity of the durotomy. One report found the incidence of durotomy to be 16%, although no long-term sequelae were noted [9]. Because exposure with the tubular retractor systems produces minimal “dead space,” the risk of postoperative dura-cutaneous fistula is reduced with tubular retractor-based surgery in comparison to traditional laminectomy. Small, stable tears may be successfully managed with a small pledget of a hemostatic agent followed by a dural sealant (e.g., fibrin glue). Larger tears or tears with exposed nerve root should be treated with direct suture repair. Although technically demanding, this can be achieved using a small needle and micropituitary instrument as the needle driver and an arthroscopic knot pusher to assist with knot typing. In most cases, prolonged bed rest is not required for patients after a satisfactory dural repair [18].

Infection rates following tubular access surgery are low [19]. In the rare event of a wound infection, treatment with debridement and antibiotic therapy should be instituted. Due to the lack of prolonged anesthesia, heavy blood loss and prolonged bed rest, medical complications after tubular access decompression, are uncommon even in the elderly population [2].

## 8. Conclusion

With the use of a tubular retractor system and microscope, lumbar stenosis can be successfully treated in the majority of patients. This approach has significant advantages when compared to traditional laminectomy including reduced blood loss, reduced hospitalization, reduced infection, and quicker postoperative recovery. As with all new surgical techniques, an operative learning curve should be anticipated. The learning curve may be successfully managed by supervised cadaver training, surgical visitations and/or formal surgical mentorship. Additionally, it is recommended that the surgeon proceed in a slow, deliberate fashion from simple to more complex cases. Outcome studies have consistently documented favorable results with tubular-based decompression surgery, making this technique worth adding to a surgeon's repertoire.
